# Supplementary material for: Splice-Junction-Based Mapping of Alternative Isoforms in the Human Proteome
Source: Cell Rep. Author manuscript; Available in PMC 2020 Jan 15. (PMC6961840; doi:10.1016/j.celrep.2019.11.026)
Supplement: 2 [file NIHMS1546469-supplement-2.zip › DF1/PXD010154/Heart/Heart_34_TTN_DFKTHANLFVKAPHVEFLRPLTDLQVR.pdf]

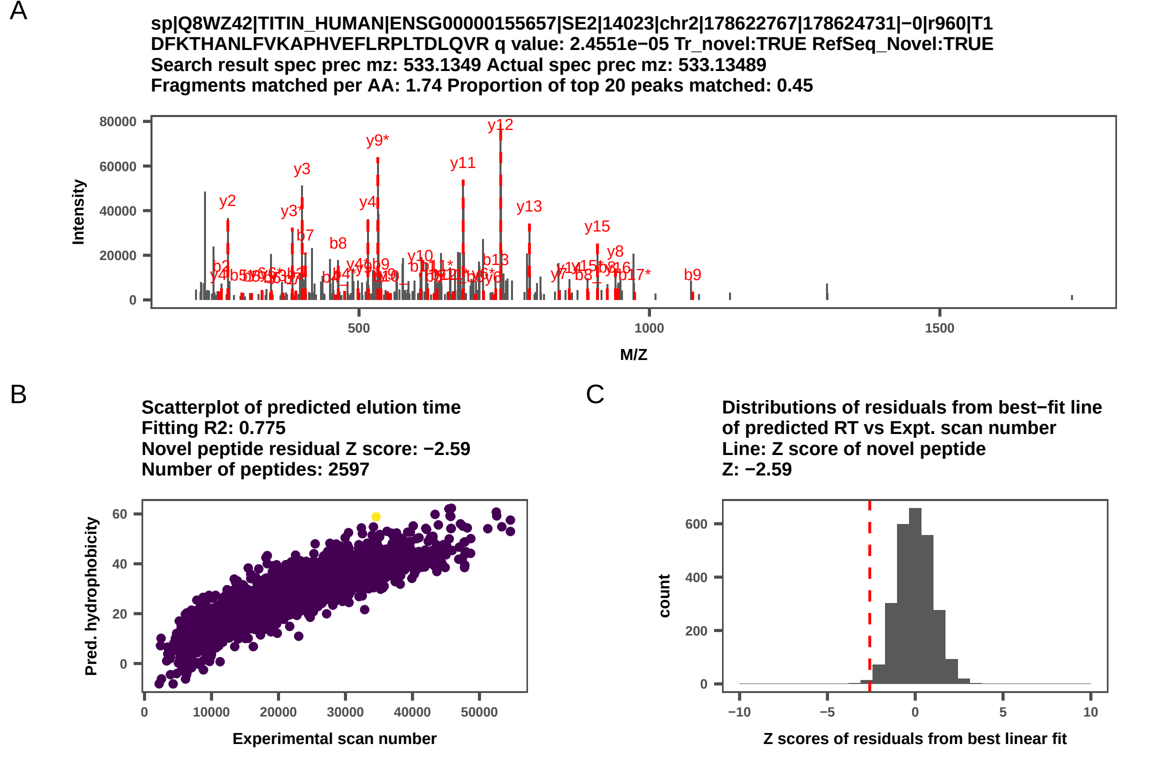
A
sp|Q8WZ42|TITIN_HUMAN|ENSG00000155657|SE2|14023|chr2|178622767|178624731|−0|r960|T1
DFKTHANLFVKAPHVEFLRPLTDLQVR q value: 2.4551e−05 Tr_novel:TRUE RefSeq_Novel:TRUE
Search result spec prec mz: 533.1349 Actual spec prec mz: 533.13489
Fragments matched per AA: 1.74 Proportion of top 20 peaks matched: 0.45
Intensity
80000
60000
40000
20000
0
M/Z
500
1000
1500
y2
y3
y3*
y4
y9*
y11
y12
y13
y15
y8
b7
b8
b9
b13
y10
b2
B
Scatterplot of predicted elution time
Fitting R2: 0.775
Novel peptide residual Z score: −2.59
Number of peptides: 2597
Pred. hydrophobicity
60
40
20
0
Experimental scan number
0
10000
20000
30000
40000
50000
C
Distributions of residuals from best−fit line
of predicted RT vs Expt. scan number
Line: Z score of novel peptide
Z: −2.59
count
600
400
200
0
−10
−5
0
5
10
Z scores of residuals from best linear fit
